# ﻿Taxonomy of the weed species of the genus *Echinochloa* (Poaceae, Paniceae) in Southwestern Europe: Exploring the confused current state of affairs

**DOI:** 10.3897/phytokeys.197.79499

**Published:** 2022-05-23

**Authors:** Ivan Hoste, Filip Verloove

**Affiliations:** 1 Meise Botanic Garden, Nieuwelaan 38, B-1860 Meise, Belgium Meise Botanic Garden Meise Belgium

**Keywords:** *Echinochloa*, evolutionary history, lectotypification, nomenclature, Poaceae, Southwestern Europe, taxonomy

## Abstract

The taxonomy of *Echinochloa*, a predominantly tropical to warm-temperate genus of 40–50 species, including some of the world’s worst weeds, is still poorly understood. This is because some species, including the extremely widespread *E.crus-galli*, show a wide range of morphological, physiological and ecological variation, in part the result of a complex recent evolutionary history. Furthermore, there is often a dearth of clear distinguishing features among species. The same applies to the species established in Southwestern Europe, where unintentionally introduced populations have now established themselves as important weeds of crops, especially maize and rice. Taxonomic and nomenclatural confusion hampers progress in weed science. In this study, we give an identification key that covers the weedy taxa encountered in Southwestern Europe, followed by notes on taxonomy and nomenclature. Moreover, a lectotype is designated for *Echinochloafrumentacea*. It is argued that current confusion cannot be overcome without including populations of Eastern Asian origin in taxonomic studies and without the joint efforts of experts in the fields of weed science, morphology-based taxonomy, genomics and phylogenetics.

## ﻿Introduction

*Echinochloa* P. Beauv. is a predominantly tropical to warm-temperate genus of 40–50 species that are usually associated with wet or damp places (Michael 2003). *Echinochloacrus-galli* (L.) P. Beauv., by far the most widespread species of the genus, is among the worst weeds worldwide (Holm et al. 1977). However, in Western and Southern Europe, *E.crus-galli* is not the only troublesome species of this genus of Poaceae. Together with several other C_4_ grasses of the genera *Digitaria* Haller, *Panicum* L. and *Setaria* P. Beauv., *Echinochloamuricata* (P. Beauv.) Fernald, too, has become a widespread weed, especially in maize fields, in the past few decades (Jauzein and Montégut 1983; Scholz 1995; Hoste 2004). Furthermore, the spread of a few additional taxa of Asian origin in rice fields in the Mediterranean area has increasingly challenged the identification skills of botanists and agronomists (Jauzein 1993; Viggiani and Tabacchi 2017; Martínez-Azorín and Crespo 2021).

The taxonomy of *Echinochloa* is still poorly understood, resulting in strongly diverging interpretations of its classification and nomenclature. These divergent interpretations can be attributed to several reasons, such as the wide range of within-species variation (not in the least in ill-defined and polymorphic *E.crus-galli*), the recurrent absence of unequivocal qualitative and quantitative distinguishing features among species, insufficient joint research by taxonomists and agronomists and the often extended lag time between the introduction of an exotic taxon in a new geographic region and its detection and correct identification by local botanists and weed scientists. As a result of the description of numerous taxa with probably little or no taxonomic value, quite a few species may be overvalued.

In Southwestern Europe, taxonomically widely divergent treatments of the genus *Echinochloa* are available for the British Isles (Hubbard 1968; Cope and Gray 2009; Stace 2019), the Netherlands (Duistermaat 2020), Belgium (Lambinon and Verloove 2012; Verloove 2021), France (Jauzein 1993, 1995; Tison and de Foucault 2014), Central Europe (Conert 1998; Parolly and Rohwer 2019), the Iberian Peninsula (Martínez-Azorín and Crespo 2021) and Italy (Pirola 1965; Pignatti 1982; Viggiani et al. 2003; Banfi 2017; Viggiani and Tabacchi 2017). Nothing better illustrates the confusion and changing views on taxonomy and nomenclature of *Echinochloa* in Southwestern Europe during the past half-century than the five references given for Italy. The tangled web of confusion is also revealed in a quote by Tabacchi et al. (2006) about Early watergrass (*E.oryzoides*) as “never been reported before in Italy,” whereas the species was described (as *Panicumoryzoides*) on the basis of material that was in all likelihood collected in Italy (see below).

In an overview of the weedy species of *Echinochloa* in Southwestern Europe, Carretero (1981) concentrated on presence in rice fields in Italy, Southern France, Spain and Portugal. He mentioned two indigenous species, *E.crus-galli* and *E.colona* (L.) Link, plus three introduced taxa of Asian origin. Two decades later, Costea and Tardif (2002), in a paper on “the most common weedy European *Echinochloa* species,” never mentioned *E.muricata*. However, by then, this American species had been recorded as a weed from Camargue, France, and Jauzein (1993) urged botanists to be watchful of *E.muricata*, which he warned had recently started spreading quickly in other parts of France. Unfortunately, to date, this species has hardly ever been mentioned in botanical and weed science papers dealing with Southern Europe: although the species is definitely not common, it may have been overlooked. There is also doubt about its status in the British Isles. Cope and Gray (2009) claimed that some races of *E.crus-galli* “have been considered worthy of recognition at species level, but there is no general agreement on this.” *Echinochloamuricata* is not included in the keys by Stace (2019), yet the author observed that some specimens keying out as *E.crus-galli* would belong to E.muricatasubsp.microstachya (Wiegand) Jauzein.

Distinguishing between American *E.muricata* and European *E.crus-galli* based on morphology is relatively easy, yet separating the latter from persistent and morphologically variable *Echinochloa* introduced from Asia and today thriving in rice fields in Southern Europe proves much more difficult. The contrasting treatments of *Echinochloa* in Japanese (Ohwi 1965; Ibaragi 2020) and Chinese (Shouliang 1990; Shouliang and Phillips 2006) floras only accentuates the confusion.

To develop superior control methods in crops, including rice and maize, basic knowledge of the classification, morphology, physiology and ecology of specific weeds is essential (Yabuno 1983). Recent advances in molecular techniques have created new opportunities to study the weedy species of genus *Echinochloa*. New research combining morphological and molecular data has been undertaken with the aim to better understand the species’ classification and establish useful morphological traits that allow weed scientists and farmers to reliably identify the different taxa. To date, it has been shown that *E.muricata* and *E.crus-galli* are clearly distinct (Claerhout et al. 2016); however, studies dealing with the taxa of Asian origin and specifically aspiring to integrate morphological and molecular data have, so far, yielded only limited success (e.g., Yasuda et al. 2002; Yamaguchi et al. 2005; Ruiz-Santaella et al. 2006; Tabacchi et al. 2006; Aoki and Yamaguchi 2008; Lee et al. 2014a, 2014b; Ye et al. 2014; Yasuda and Nakayama 2019). Often with *E.oryzicola* (Vasinger) Vasinger as the exception, matching the data from genetic research with the multitude of names and descriptions from the morphology-based literature remains ridden with difficulties. Nomenclatural confusion resulting in the same name being applied to different taxa in different studies is a source of uncertainty and may render the interpretation of published research results precarious, especially when no herbarium specimens have been deposited (Yamaguchi et al. 2005). Moreover, the naming of specimens based on the two different and widely diverging identification keys from Carretero (1981) and Pignatti (1982) has also not been helpful to link molecular data with morphology-based taxa (Tabacchi et al. 2006; Kaya et al. 2014). Claerhout et al. (2016) warned that using incorrectly identified seeds accessed from institutes or companies in experiments is a potential source of errors. This probably explains the position of ‘*E.muricata*’ among a cluster of *E.crus-galli* accessions in the phylogenetic tree proposed by Lee et al. (2016; fig. 2). For the same reason, an accession from a Spanish rice field (Seville) identified as ‘*E.crus-pavonis*’ (Ruiz-Santaella et al. 2006) seems doubtful as this species is not mentioned by Martínez-Azorín and Crespo (2021) and is probably not present as a weed in rice fields anywhere in Southern Europe (Michael 1983).

Morphology-based distinguishing traits frequently used in keys and descriptions often find no confirmation in molecular data. An attempt to bridge the gap with a modified “simple and effective morphological key” (Tabacchi et al. 2006) was not convincing and has been replaced later with a highly modified version (Viggiani and Tabacchi 2017). Most of the authors dealing with the problem declare a stalemate and put their hopes in future research. With this paper, we do not have the ambition to resolve the taxonomic and nomenclatural puzzle posed by *Echinochloa* in Southwestern Europe. Instead, our goal is twofold. On the one hand, we present a provisional key that makes it possible to identify the weedy species occurring in Southwestern Europe (from the British Isles to Portugal and Italy); critical comments are added to explain our choice of accepted taxa. On the other hand, we wonder why matching the results of recent molecular studies on *Echinochloa* in Europe and the Far East with those obtained by morphological research is so problem-ridden. The current variation of taxa in the genus *Echinochloa*, including some that were recently inadvertently introduced to Southwestern Europe, is partly the result of a complex evolutionary history, the traces of which are visible in the morphological and genetic characteristics of currently existing taxa. We include the timescales of both geological epochs and human history to frame the future study of the taxonomy and phylogeny of weedy *Echinochloa* in Southwestern Europe.

## ﻿Result

### ﻿An identification key for the species of *Echinochloa* in Southwestern Europe

Identification keys for *Echinochloa* in floras or weed science papers are often restricted to a rather small geographical area. Covering a larger area and more taxa may lead to more attention being paid to taxa which, so far, could have been overlooked. As far as the reviewed European literature is concerned, this paper is mainly restricted to Southwestern Europe, roughly stretching from the British Isles in the north to the Iberian Peninsula and Italy in the south. The key should, however, prove useful to identify the established weedy species of the genus *Echinochloa* in most of Europe. Owing to nomenclatural and taxonomic uncertainties, the key is considered provisional; for a different recent interpretation, see Martínez-Azorín and Crespo (2021).

A number of rare casuals that have been reported from Europe in the past, for instance, as wool aliens, have been omitted. These include *Echinochloainundata* Michael & Vickery and *E.jubata* Stapf from Belgium (Verloove 2021), *E.turneriana* (Domin) J.M.Black from Germany (Conert 1998) and *E.crus-pavonis* (Kunth) Schult., *E.pyramidalis* (Lam.) Hitchc. & Chase (a perennial species) and *E.turneriana* from Great Britain and Ireland (Ryves et al. 1996; Reynolds 2002). Adding these species – the exact identity of some of which requires confirmation – would have made the key unnecessarily difficult. Moreover, there is currently no indication for these ephemerals establishing as troublesome weeds in crops.

*Echinochloacrus-pavonis* has been excluded from the key since the records from rice fields in Southern Europe seem to be based on erroneous identifications (Banfi 2017); the photographs given by Viggiani et al. (2003, pages 242–243) show a form of *E.crus-galli* s.l.

Those who run into problems when using the key given below or suspect they are dealing with a species missing from the key are referred to the keys to the annual and perennial species of *Echinochloa* produced by P.W. Michael (1983), with updates, including those from Michael (2019). In Europe, the known weedy species are all annuals.

In combination with the wide variation within individual species, the dearth of strong qualitative and quantitative features precludes easy identification in the genus *Echinochloa*. Within the same inflorescence, the spikelets may show considerable variation. The number, size, position and direction of hairs and bristles is often strongly influenced by competition for space among the closely packed spikelets. The length of the lower glume and the shape of the sterile lemma (occasionally part of them shiny and convex) can be assessed only by examining several spikelets. The length of the spikelet – excluding the awn of the sterile lemma – is an important feature (Michael 1983; Jauzein 1993). Especially when awned or having an elongated tip, measuring the length of the spikelet may prove difficult as deciding where the spikelet passes into the awn is rather arbitrary. The presence of spikelets in which the upper glume has an elongated tip or a short awn (as sometimes occurs in several taxa) renders a correct measurement more uncertain.

**Table:** 

1	Fertile floret not disarticulating at maturity. Spikelets unawned. Fertile floret and caryopsis markedly humped. Inflorescence compact, usually contracted and with the axis often hardly visible, sometimes with spreading branches (Fig. 1)	**2**
1'	Fertile floret disarticulating at maturity. Spikelets awned or not. Fertile floret and caryopsis not markedly humped. Inflorescence not strongly contracted when fully developed, with the axis showing through (but compare with clearly different E.muricatavar.wiegandii when in doubt)	**3**
2	Spikelets dark brownish or purplish at maturity (Fig. 2), ca. 3–4 mm long. Caryopsis brownish	***E.esculenta***
2'	Spikelets pale (yellowish or greenish) at maturity (Fig. 1), ca. 3–3.5 mm long. Caryopsis whitish	***E.frumentacea***
3	Spikelets < 3 mm long and lower glume ca. 1/2 length of the spikelet, which is always unawned. Axis of the inflorescence branches (almost) without bristles (except at the base). Inflorescence without secondary branches (Fig. 3). Leaves narrow, usually not exceeding 6 mm. Caryopsis whitish	***E.colona***
3'	Spikelets usually ≥ 3 mm long, awned or not. (If spikelet < 3 mm, then lower glume only ca. 1/3 length of the spikelet.) Axis of the inflorescence branches with bristles. Inflorescence often with secondary branches. Leaves usually wider. Caryopsis usually darker, yellowish or brownish	**4**
4	Spikelets ≥ 4 mm long and at least some spikelets with lower glume up to 2/3 length of the spikelet (Fig. 4D). Mature inflorescence more or less erect (Fig. 5). Spikelets unawned or with an awn up to 20 mm long. Caryopsis 2–2.4 mm long. Embryo at least 0.75 to over 0.9 length of the caryopsis. (An obligate weed of rice.)	***E.oryzicola***
4'	Spikelets ≥ 4 mm long and lower glume not longer than 1/2 length of the spikelet. Mature inflorescence drooping (Fig. 6). Spikelets usually awned, with an awn up to 50 mm long. Caryopsis 2.2–2.8 mm long. Embryo 0.65–0.75(–0.85) length of the caryopsis. (An obligate weed of rice.)	**E.crus-gallivar.oryzoides**
4"	Spikelets ≤ 4 mm long and lower glume usually clearly less than 1/2 length of the spikelet. (If spikelets > 4 mm, see 6, E.muricatavar.muricata. Solely a rare casual?)	**5**
5	Lemma of the fertile floret with a membranous tip that is clearly differentiated from the coriaceous body of the lemma (Fig. 7A); the membranous tip demarcated from the coriaceous body by a line of minute hairs (the latter, however, not or hardly visible with a hand lens). Palea of the fertile floret with a blunt, soft, frayed looking, usually strongly recurved tip (Fig. 7B). Spikelets unawned or awned; awn length extremely variable (up to 40 mm long or more). The leaf subtending the distal inflorescence with the demarcation between blade and sheath more or less semicircular or forming a slightly elongated upside-down U; blade usually patent from the base. (A complex taxon with several difficult-to-distinguish intergrading forms that are not keyed out here; see comments below.)	***E.crus-galli***
5'	Lemma of the fertile floret with a stiff, smooth tip, not clearly differentiated from the coriaceous body of the lemma (Fig. 8A). Palea of the fertile floret with a stiff, (nearly) straight tip; the tip (in mature florets!) appressed against the lemma (Fig. 8B). Spikelets unawned or awned, with the awn usually shorter than 10 mm (but longer in the rare var. muricata). The leaf subtending the distal inflorescence with the demarcation between blade and sheath forming an elongated upside-down U (Fig. 9); blade stiff upright (esp. when short) or recurved higher up (***E.muricata***)	**6**
6	Spikelets ≤ 3.5 mm long, with strongly spreading papilla-based bristles (which give the spikelet a rugged appearance), unawned or at most with an elongated tip (Fig. 10). Tip of both the lemma and palea of the fertile floret short. Inflorescence often large (not uncommonly > 20 cm long), when mature with widely spreading lower branches	**E.muricatavar.microstachya**
6'	Spikelets ≤ 3.5 mm long; the papilla-based bristles not strongly spreading. Numerous spikelets in the inflorescence with a short awn (sometimes up to ca. 10 mm) (Fig. 11). Tip of the palea of the fertile flower fine and elongated, fitting with the elongated tip of the lemma. Inflorescence usually smaller, its branches usually not spreading when mature	**var. wiegandii**
6"	Spikelets ≥ 3.5 mm long; numerous spikelets in the inflorescence with a longer awn (up to 16 mm). (Apart from the presence of awns, the rugged spikelets look like a more robust version of var. microstachya.) (Probably only a rare casual.)	**var. muricata**

### ﻿Notes on the species included in the key

#### ﻿ Echinochloa colona

Taxon classificationPlantaePoalesPoaceae

(L.) Link, Hort. Berol. 2: 209. 1833.

6409679F-4BF1-53E0-AFC2-118BC0C5D3F3

##### Basionym.

*Panicumcolonum* L., Syst. Nat. (ed. 10) 2: 870. 1759.

##### Type.

LINN-80.23 (lectotype, designated by Hitchcock 1908). Image available at http://linnean-online.org/1255/.

##### Remarks.

*Echinochloacolona* is usually easy to identify, yet care should be taken to distinguish it from forms with small spikelets of *E.crus-galli* (Martínez-Azorín and Crespo 2021). In the Mediterranean region, it occurs as a persistent weed in crop fields; elsewhere, it has only been recorded as a usually ephemeral alien.

#### ﻿ Echinochloa crus-galli

Taxon classificationPlantaePoalesPoaceae

(L.) P. Beauv., Ess. Agrost. 1: 53, 161, 169, pl. 11, f. 2. 1812.

9F364C56-4E9A-5FF6-9C22-06BF010192CA

##### Basionym.

*Panicumcrus-galli* L., Sp. Pl. 1: 56. 1753.

##### Type.

Herb. Burser 1: 303, sine dato (UPS).

##### Notes.

There has always been a great deal of confusion about the type; see, e.g., Hitchcock (1908) or Gould et al. (1972). In fact, all original material came from North America and belongs to *E.muricata*; in 1753 *E.crus-galli*, from Eurasia, was not yet a widespread introduced species in North America. Crespo et al. (2020b) formally proposed to conserve the binomial *P.crus-galli* with a conserved type based on the specimen Herb. Burser I: 103 (UPS), the one previously chosen as “lectotype” by Michael (1983).

#### ﻿ Echinochloa crus-galli var. crus-galli

Taxon classificationPlantaePoalesPoaceae

53ECE520-9499-5876-89AB-5309CDE5E1C9

= Echinochloa crus-galli subsp. spiralis (Vasinger) Tzvelev, Zlaki SSSR 662. 1976. Basionym: Echinochloaspiralis Vasinger, Flora SSSR 2: 739–740. 1934. Type: Caucasus: Kuban: Krasnodar vic., 28 Oct 1931, *A.V. Vazinger-Alektorova* s.n. (holotype; LE).
= Echinochloa crus-galli var. praticola Ohwi, Acta Phytotax. Geobot. 11: 37 1942. Type: Kiushiu, m. Kujusan, *U. Faurie* 2646 (holotype; KYO). Image available at http://www.museum.kyoto-u.ac.jp/collection/PlePlant/PlePlant00001775_1.htm.
= Echinochloacrus-gallivar.hispidula (Retz.) Honda, Bot. Mag. (Tokyo) 37: 122. 1923.Basionym:Panicumhispidulum Retz., Observ. Bot. 5: 18. 1789. Type: India: “India orientali”, without data, *König* s.n. (LD 1219266) (lectotype, designated by Fischer 1932: 71). Image available at https://plants.jstor.org/stable/10.5555/al.ap.specimen.ld1219266.
= Echinochloaerecta (Pollacci) Pignatti, Arch. Bot. 15(1): 2. 1955. Basionym:Panicumerectum Pollacci, Atti Ist. Bot. Univ. Pavia 13: 228, t. 5. 1908. Type: Italy: Lombardia, Presso Pavia, Oct 1907, *G. Pollacci* s.n. (lectotype, designated by Ardenghi et al. 2015: 135, PAV-Erbario Lombardo “118”, isolectotypes PAV-Erbario Lombardo “121”, “141”, “123”, “137” (2 sheets), “139”, “140”).

#### ﻿ Echinochloa crus-galli

Taxon classificationPlantaePoalesPoaceae

var. oryzoides (Ard.) Lindm., Svensk Fanerogamflora 69. 1918.

BCCF9FB0-E286-50ED-8541-D24643A5368A

##### Basionym.

*Panicumoryzoides* Ard., Animadv. Bot. Spec. Alt. 2: 16, pl. 5. 1764.

**Figure 1.**
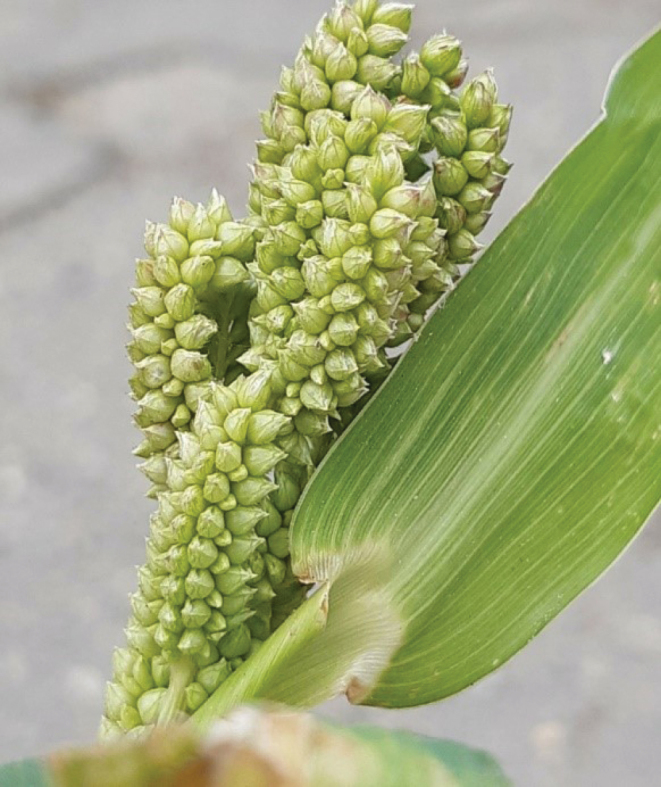
Inflorescence of *Echinochloafrumentacea*. (Photograph: Nico Wysmantel).

**Figure 2.**
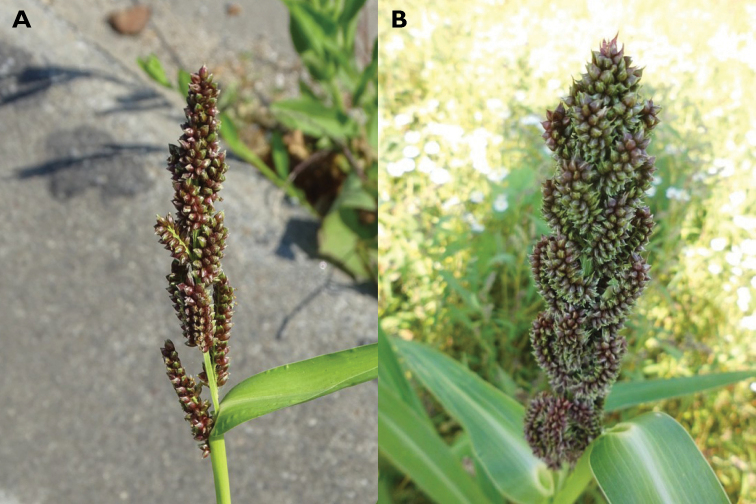
Inflorescences showing the variation of *Echinochloaesculenta*. The apex of the spikelet varies from usually obtuse (**A**) to less often shortly acute (**B**). (Photographs: Bart Mortier).

##### Type.

LINN 80.68. Image available at https://linnean-online.org/1302/.

##### Note.

According to Carretero (1981), LINN 80.68 is a plant sent by Arduino to Linnaeus, possibly collected in Italy. It was designated as the lectotype for that name by Crespo et al. (2020a).

##### Remarks on *E.crus-galli*.

*Echinochloacrus-galli* s.l. is taxonomically the most complex *Echinochloa* occurring as a weed in Southwestern Europe. As we understand, this species occurs in a number of varieties, but *E.oryzicola* is not one of them and is accepted as a separate species (see below). As a result of a long and complex evolutionary history, including significant modifications in the recent past (after the introduction of agriculture), the differences among the varieties are often slight. Furthermore, introductions of several taxa as weeds in a range of crops far outside their natural range have contributed to obscuring their original geographical distribution. Rather than aiming at precisely describing the limits and defining features of varieties of *E.crus-galli* occurring in Southwestern Europe, we restrict ourselves primarily to indicating where unsolved problems remain.

Being extremely polymorphic, numerous varieties of *E.crus-galli* have been described, many of them based on the presence or absence of awns. As the development of awns is influenced by environmental conditions (Michael 1983), the value of varieties or forms based on such characteristics as the presence or absence or the length of awns is quite limited. Inflorescences that develop later in the season frequently differ from the terminal inflorescence. Other characteristics on which the description of varieties has been based include the coloration of the plant (inflorescence, leaves, stem nodes, etc.), structure and position of the inflorescence (erect, bent or nodding; primary branches more or less patent or not, alternately positioned on the main axis or whorled), the arrangement of the spikelets on the branches and the dimensions of the spikelet. The importance of the length of the spikelets is emphasised by Michael (1983), who in his identification key for the annual *Echinochloa* separates the species characterised by spikelets measuring 3–5 mm from those with either shorter or longer spikelets. Applied to specimens collected in Southwestern Europe, the criterion of spikelet length works well to separate only the two rice mimics, E.crus-gallivar.oryzoides (Ard.) Lindm. (syn.: *E.oryzoides* [Ard.] Fritsch) and *E.oryzicola*, from the remaining taxa of *E.crus-galli* s.l. with smaller spikelets.

There is a broad consensus that E.crus-gallivar.crus-galli occurs in large parts of Europe and Asia, but authors differ on how to appropriately define it. Ibaragi (2020) stated that Asian var. crus-galli slightly differs from plants in Europe, “but the differences are difficult to formally distinguish.” Thus, the need for additional research on the morphological and genetic variation of the type variety throughout its range is evident.

**Figure 3.**
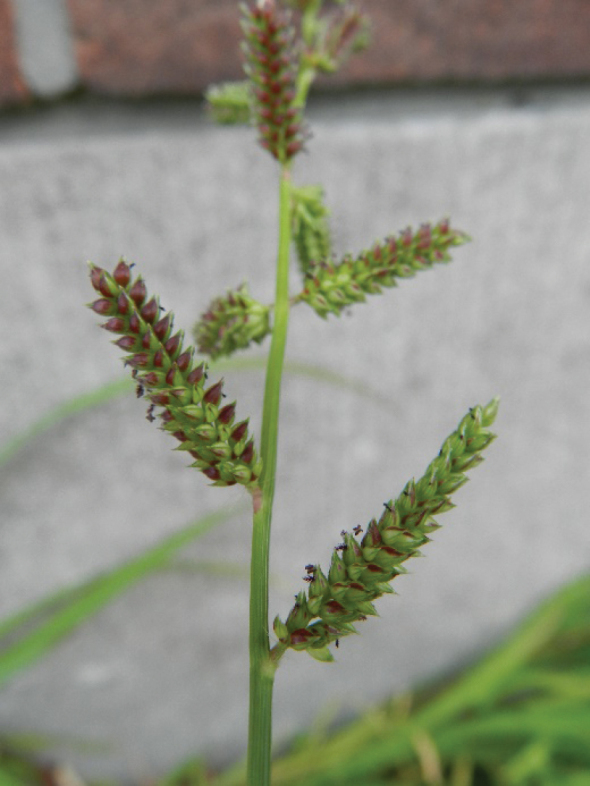
Inflorescence of *Echinochloacolona*. (Photograph: Rutger Barendse).

According to Michael (2019), Echinochloacrus-gallivar.hispidula (Retz.) Honda is the appropriate name for *E.crus-galli* with non-pyramidal panicles and usually prominently awned spikelets that are widespread in sub-tropical areas of Japan and Southern China. With slightly larger spikelets than var. crus-galli, this taxon is often treated as a separate species, *Echinochloahispidula* (Retz.) Nees ex Royle; however, Ibaragi (2020) completely ignored it, and Shouliang and Phillips (2006) interpreted it as synonym of var. crus-galli. Its extreme variability (Carretero 1981, as *E.hispidula*) makes it hard to distinguish var. hispidula from var. crus-galli, which is characterised by a usually more or less procumbent habit (the lower nodes often rooting), floppy leaves, the whole plant or parts of it more often than not purple-tinged, erect to strongly bent pyramidal inflorescences with the branches alternately placed or sometimes whorled, with at least the lower branches usually more or less patent, and spikelets with or without awns, the length of the awns and the percentage of awned spikelets within a single inflorescence exhibiting considerable variation (Fig. 12; description based on material from maize fields in Belgium, where var. hispidula, a taxon of sub-tropical climates, is considered not established as a persistent weed). If accepted as a separate taxon, the strongly bent inflorescence with appressed branches, the green colour of the plants and the stiffer leaves (the latter two features shared with var. oryzoides and *E.oryzicola*) might help to separate *E.hispidula* from E.crus-gallivar.crus-galli (Jauzein 1993). To this could be added the less bristly spikelets of var. hispidula (Martínez-Azorín and Crespo 2021). Whether the branches of the inflorescence are whorled or not (Michael 1983) seems to be a less reliable trait to use. The synonymizing of *E.erecta* (Pollacci) Pignatti, characterised by an erect inflorescence, with *E.hispidula* (see, e.g., Ardenghi et al. 2015) underscores the wide morphological variation of *hispidula* and the difficulty to define it as a unit clearly different from the equally variable var. crus-galli. Interestingly, the illustration of *E.hispidula* given in Shouliang (1990) shows a spikelet with both the upper glume and lower (sterile) lemma with a short awn, a feature not mentioned in the recent literature; however, apparently, it corresponds with the specification “calycibus hispidis biaristatis” in the original description of the species (Retzius 1789).

**Figure 4.**
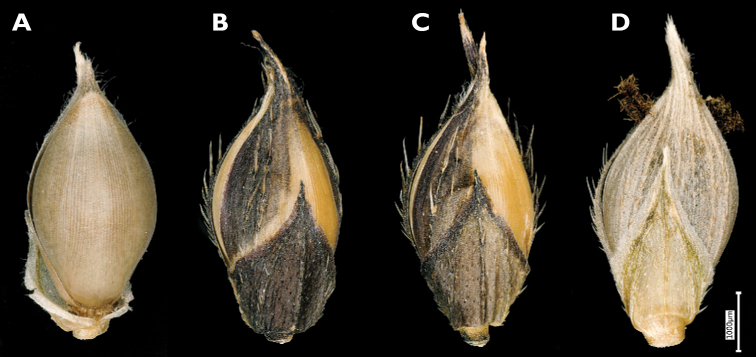
Spikelets of *Echinochloaoryzicola***A** fertile lemma with the tip differentiated from the coriaceous body of the lemma (upper glume removed) **B, C** two spikelets with convex shiny sterile lemma **D** spikelet with long lower glume and non-shiny sterile lemma. (Photograph: André De Kesel, Meise Botanic Garden).

Within E.crus-galli as interpreted here, var. oryzoides is the most easily identified variety, clearly distinguished by the large size of its spikelets. Although the descriptions given in triplet 4 in the key above may suggest otherwise, it is not always easy to distinguish between var. oryzoides and *E.oryzicola*; see the discussion about the latter species below. At one time, the name *Echinochloahostii* (M. Bieb.) Link was used by Italian botanists (Pignatti 1982). Previously, Pirola (1965) merely cited this name as a synonym of E.crus-gallisubsp.oryzoides, but Pignatti (1982) accepted the name at species rank for the taxon that is here named E.crus-gallivar.oryzoides. However, from Pignatti’s identification key, it is clear that the name *E.hostii* was used for the species that today can only be identified as *E.oryzicola*, based on the quite diagnostic glume characteristics. Unfortunately, we were not able to trace type material of *Panicumhostii* M. Bieb. which according to Tsvelev (1984) is preserved in LE. Thus, we do not know whether *P.hostii* is indeed identical with E.crus-gallivar.oryzoides as stated by nearly all contemporary authors. Nonetheless, we are certain that the binomial *E.hostii* was wrongly applied by Italian authors (particularly Pignatti 1982) for *E.oryzicola*.

**Figure 5.**
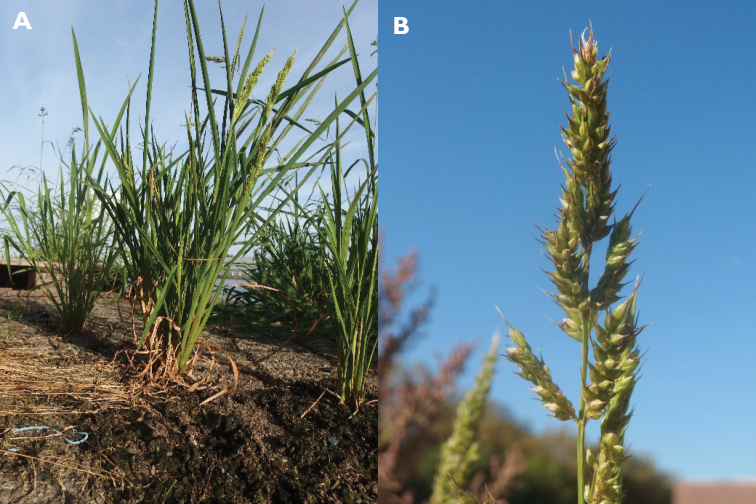
*Echinochloaoryzicola* as an ephemeral casual in the Antwerp port area, Belgium **A** plant with young inflorescences **B** part of an inflorescence with fully developed spikelets. (Photographs: Filip Verloove).

Recently, Martínez-Azorín and Crespo (2021) accepted E.crus-gallivar.oryzoides as a species, just like the similar-looking *E.oryzicola*. The strong similarities shared by these two taxa are explained by a shared ancestor – tetraploid *E.oryzicola* being one of the parent species of hexaploid *E.crus-galli* – and recent convergent evolution as rice mimics derived from *E.oryzicola* and *E.crus-galli* (Fig. 13). This evolutionary trajectory provides an argument for assigning the rank of variety to *E.oryzoides*. Further, it would seem logical to reduce the rice mimic *E.oryzicola* to the rank of variety (provisionally ‘var. infestans’ in Fig. 13) as well, but since it is not known whether or how the ‘original’ *E.oryzicola* of pre-agricultural times differed from today’s *E.oryzicola* – because it is now extinct or goes undetected – this is not an option.

Among the forms with small spikelets, subsp. spiralis (Vasinger) Tzvelev (no combination available as a variety) and var. praticola Ohwi have been mentioned as occurring in Europe. Apparently solely based on the small spikelets, both names were synonymised by Scholz (2002), who noted that subsp. spiralis – a taxon with a huge distribution area and possibly indigenous to Europe – and subsp. crus-galli are polymorphic and that no sharp distinction between the two is possible solely based on the spikelet length. Martínez-Azorín and Crespo (2021) interpreted var. praticola as probably no more than an impoverished form of *E.crus-galli*, and Tison and de Foucault (2014) seriously doubted the taxonomic value of subsp. spiralis and var. praticola. In Belgium also, *E.crus-galli* with small spikelets has been recorded, but nowhere have such plants been known to establish as noxious weeds.

**Figure 6.**
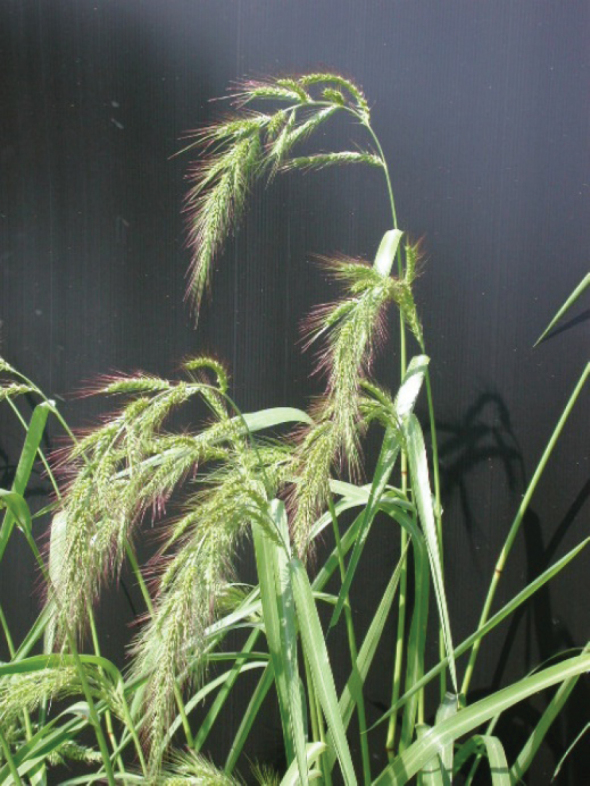
Habit of Echinochloacrus-gallivar.oryzoides cultivated from seeds collected in rice, Italy. (Photograph: Maurizio Tabacchi, ISIDRO, Italy).

A rather distinct form of *E.crus-galli* with spikelets ca. 3 mm long or a little longer (somewhat smaller than average var. crus-galli) has occasionally been observed in Belgium, including in the border of maize fields where, however, it seems not to establish easily and disappears after only a few years. These plants usually have an erect habit and rather stiff leaves. The inflorescence is erect, with patent branches. The purple-tinged spikelets are usually unawned (but a few spikelets may have a long awn), and some have a glabrous, convex and shiny sterile lemma. The lower leave sheaths vary from glabrous to densely covered with short retrorse hairs. Scholz (2002) included specimens with small spikelets with a convex, shiny sterile lemma in subsp. spiralis, mentioning that the spikelet morphology resembles *Echinochloaglabrescens* Munro ex Hook.f. Another name for *E.glabrescens* is E.crus-gallivar.formosensis Ohwi (Yabuno 1983; Ibaragi 2020); this name was used by Japanese authors for a weed of wetland rice fields. The habitats in which the plants were found in Belgium stand in contrast to those preferred by var. formosensis in Japan. Adding to the confusion, the name *E.glabrescens* has also been applied to plants with spikelets 3.5–5 mm long by Bor (1960), Shouliang and Phillips (2006; as *E.glabrescens* Kossenko) and Xia et al. (2011). *Echinochloa* with small spikelets and a shiny lower lemma collected in Europe requires more study in order to reveal its true identity and its relation with similar taxa having small spikelets in Asia and *E.oryzicola*; see, e.g., Yasuda and Nakayama (2019). Although a quite distinctive feature, the convex and shiny sterile lemma might prove to be of little value taxonomically. Bor (1960) wondered whether the “most peculiar” feature of the indurated sterile lemma in *E.glabrescens* was sufficient to make it a good species, and Yabuno (1966) indicated that in *E.oryzicola*, the convex lemma is a simple dominant characteristic (Fig. 4B, C).

**Figure 7.**
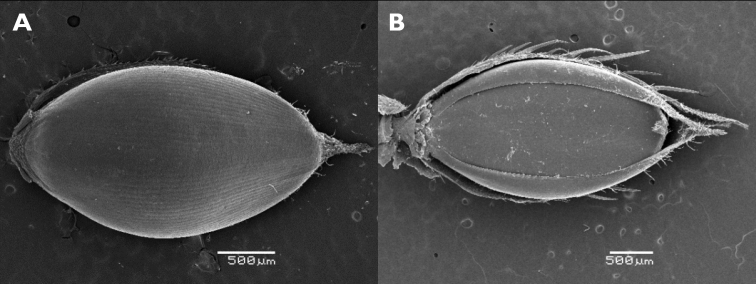
Spikelets of *Echinochloacrus-galli***A** fertile lemma with the tip differentiated from the coriaceous body of the lemma (upper glume removed) **B** spikelet with tip of the fertile palea frayed and strongly recurved (lower glume and sterile flower removed).

**Figure 8.**
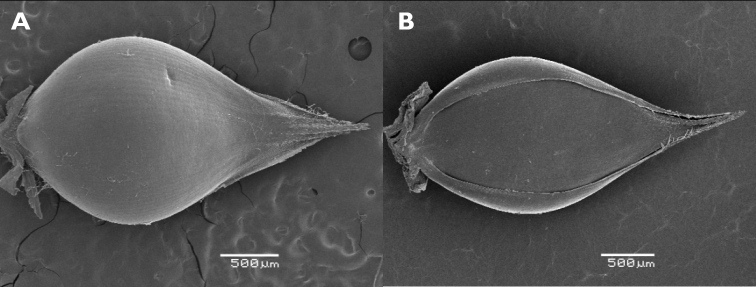
Spikelets of *Echinochloamuricata***A** fertile lemma with the tip not clearly differentiated from the coriaceous body of the lemma (upper glume removed) **B** spikelet with tip of the fertile palea stiff and straight (lower glume and sterile flower removed).

Considering the preceding discussion, we accept, for the present, only few varieties of *E.crus-galli* as occurring in Southwestern Europe. Indigenous and quite variable var. crus-galli, usually with a less erect habit and more floppy leaves, is by far the most widespread variety, especially towards the north. Part of the variation observed in Europe is perhaps due to the involuntary introduction and establishment of populations of var. crus-galli, with slightly different morphological features, from Asia. Echinochloacrus-gallivar.oryzoides, characterised by large spikelets, a more erect habit and stiffer leaves, is a rice mimic in rice fields of Southern Europe. Echinochloacrus-gallivar.hispidula , in some respects resembling var. crus-galli and in others var. oryzoides , appears to us not to deserve a separate status and is, therefore, included in var. crus-galli.

**Figure 9.**
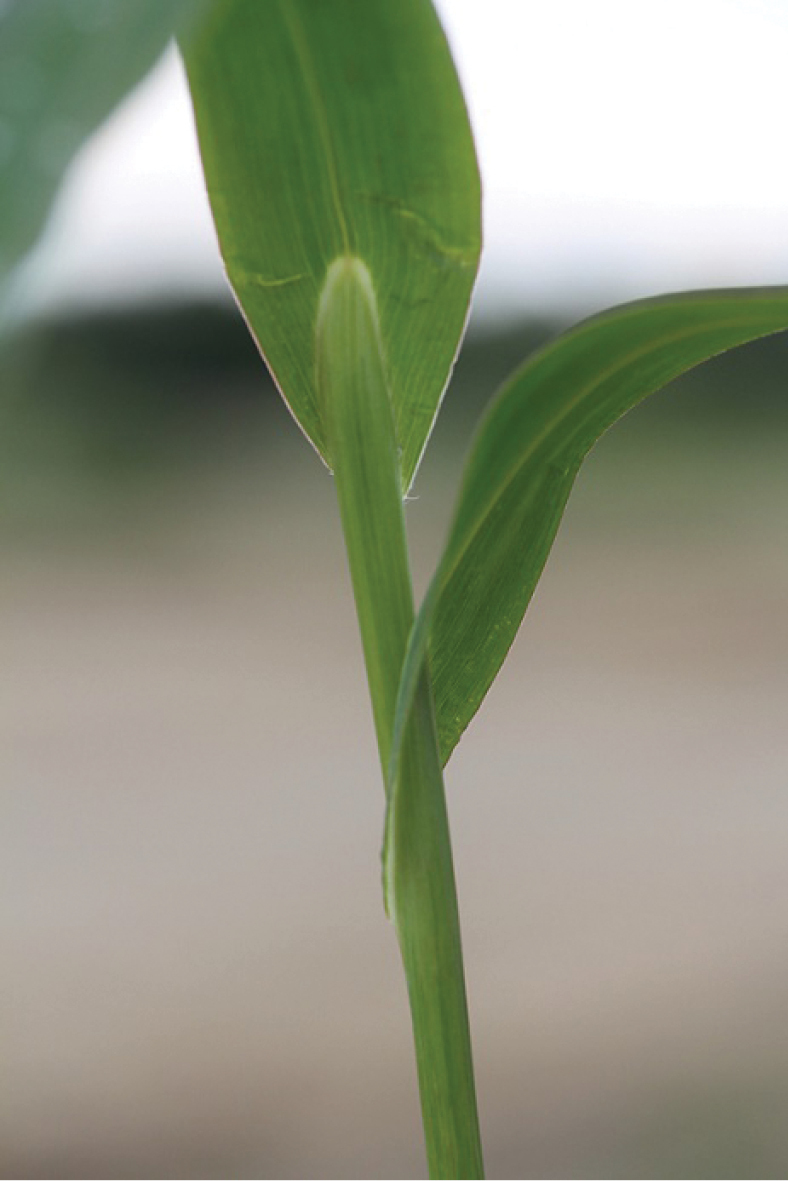
*Echinochloamuricata*. The uppermost leaf (or leaves) subtending the distal inflorescence have the demarcation between blade and sheath in the form of an elongated upside-down U. (Photograph: Rutger Barendse).

Plants with small spikelets are the most difficult to interpret. Probably representing more than one taxon – quite possibly including taxonomically irrelevant forms of var. crus-galli – they require additional study, which will need to include material of Asian origin.

Finally, it can be argued that *Echinochloaesculenta* (A. Braun) H. Scholz, a cultivated taxon derived from *E.crus-galli*, should be included in *E.crus-galli* (Banfi and Galasso 2021). Here, in line with most recent floras, it is pragmatically accepted as a well-defined separate species. However, species rank is justifiable based on morphological features, as the two taxa clearly differ from each other and identification of *E.esculenta* is usually not much of a problem.

#### ﻿ Echinochloa esculenta

Taxon classificationPlantaePoalesPoaceae

(A. Braun) H. Scholz, Taxon 41(3): 523. 1992.

EA8571BE-2D34-59A4-9DB6-A302D71793C8

##### Basionym.

*Panicumesculentum* A. Braun, Index Sem. [Berlin] 1861(App.): 3. 1861.

##### Type.

*Koernicke* s.n., Cult. Hort. Bonn-Poppelsdorf, 28 Oct 1875 (B) (neotype, designated by Scholz 1992: 523). Image available at https://ww2.bgbm.org/Herbarium/specimen.cfm?Barcode=B100366144.

##### Remark.

See the combined comments below, under *E.frumentacea*.

#### ﻿ Echinochloa frumentacea

Taxon classificationPlantaePoalesPoaceae

Link, Hort. Berol. 1: 204. 1827.

15D3E4B3-D326-598C-80C4-C97BB29D1C0D

##### Type, lectotype designated here.

India, *Roxburgh* s.n. (K000215131, the specimen on the extreme right on the sheet). Image available at http://specimens.kew.org/herbarium/K000215131.

##### Note.

The protologue refers to a Roxburgh collection from India (“Roxb. ind. 1. 307. R. S. m. 2. 250. Hab. in India orientali ubi colitur”). The Kew herbarium houses two original but undated Roxburgh collections (sheets K000215131 and K000215132) that can serve for a proper typification. None exactly matches the information provided in the protologue, but since Link described the species in 1827, i.e. well after Roxburgh’s (1751–1815) death, these collections are supposed to have been at his disposal when describing the species. In the apparent absence of other original material, one of the two above-mentioned Kew collections could be chosen as the lectotype for that name. Digital images of both are easily accessible via online resources such as the Kew Herbarium Catalogue, JSTOR or POWO. Sheet K000215131 comprises five stems, four of which have an inflorescence. The extreme left specimen is atypical and might as well represent a different species. The other flowering specimens are representative for the species, and the specimen on the extreme right is here designated as the lectotype for the name *E.frumentacea*. According to Stafleu and Cowan (1983) considerable sets of duplicates of Roxburgh specimens are stored at BM, BR, E, G and LIV. In some of these herbaria isolectotypes could thus be found although a quick online search did not yield further specimens.

##### Remarks on *E.esculenta* and *E.frumentacea*.

*Echinochloaesculenta* (syn.: *E.utilis* Ohwi & Yabuno) and *E.frumentacea* are cultivated species. Neither is considered a persistent weed in Southwestern Europe. Still, they are included in the key since they are the most frequently occurring non-weedy representatives of the genus in Southwestern Europe, frequently recorded as bird-seed aliens in and along the border of crop fields (Hanson and Mason 1985). They look similar, and young specimens can be difficult to identify, yet mature ones are easily distinguished by the colour of the spikelets. In both species, the inflorescence varies. In the more typical specimens, the branches are tightly clustered and appressed against the axis, creating a compact inflorescence. Often, however, the inflorescence is rather lax, with the distal part of the branches somewhat curved towards the axis; such specimens are easily mistaken for an awnless form of *E.crus-galli*. Yabuno (1966) describes the distinct characteristics of the two species, and recent genetic studies have confirmed that they are quite distinct, *E.esculenta* being derived from *E.crus-galli* and *E.frumentacea* from *E.colona* (Yamaguchi et al. 2005; Ye et al. 2014). There are arguments for reducing these two taxa to variety rank or, following Banfi and Galasso (2021), subspecies rank under *E.crus-galli* and *E.colona*. Yabuno (1966) insinuated that *E.esculenta* shows more variation, and Michael (1983) added that in this species, the spikelets may be awned (although awned spikelets seem to be rare); this reflects the highly polymorphic nature of the parent species.

**Figure 10.**
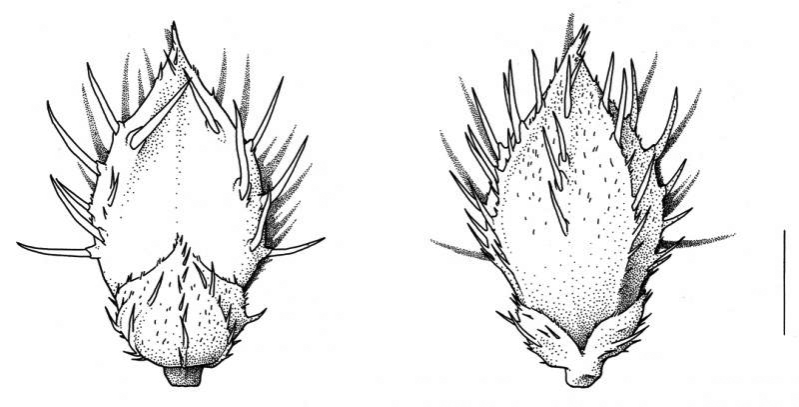
Spikelet of Echinochloamuricatavar.microstachya showing the lower glume and unawned sterile lemma (left) and upper glume (right). Scale bar 1 mm. (Drawing: Sven Bellanger, Meise Botanic Garden).

**Figure 11.**
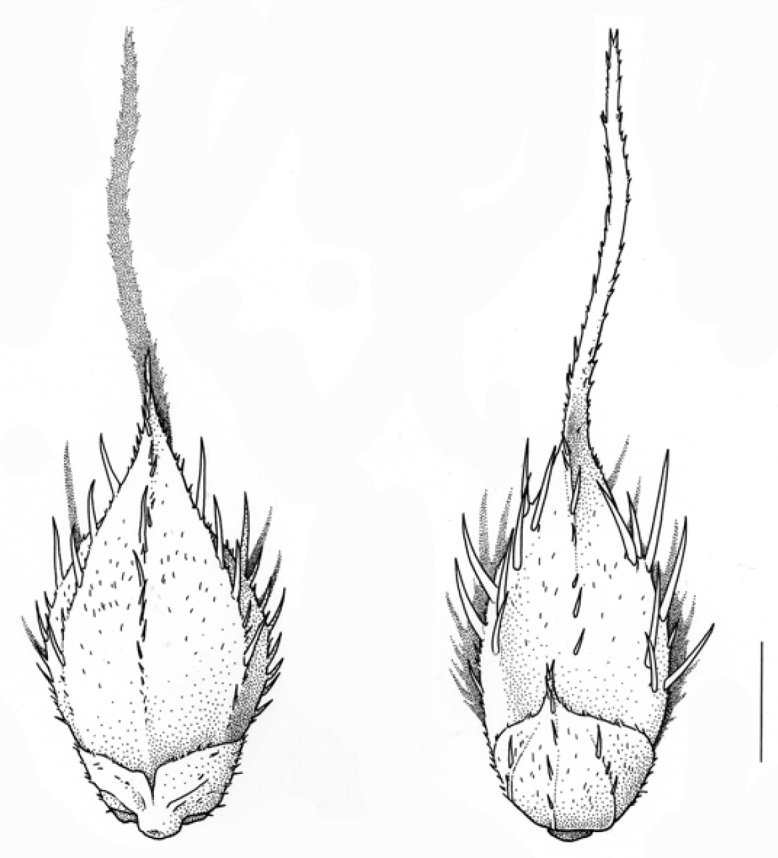
Spikelet of Echinochloamuricatavar.wiegandii showing upper glume (left) and lower glume and awned sterile lemma (right). Scale bar 1 mm. (Drawing: Sven Bellanger, Meise Botanic Garden).

**Figure 12.**
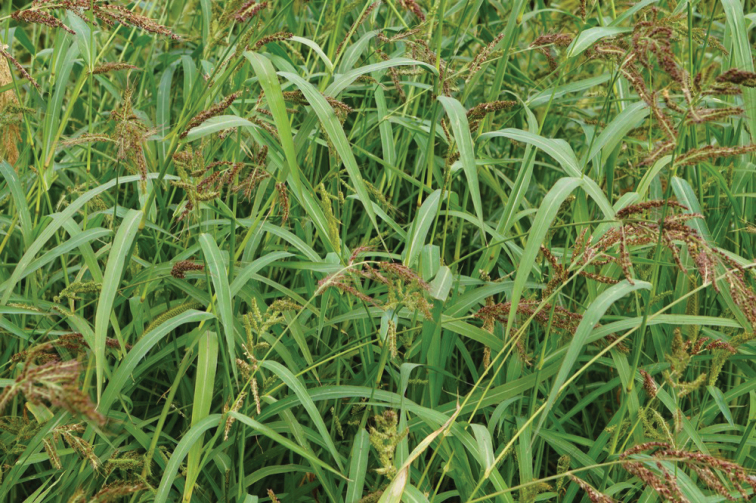
Habit of Echinochloacrus-gallivar.crus-galli growing as a roadside weed, Belgium. Although extremely variable, the usually more or less procumbent habit (the lower nodes often rooting) and the floppy leaves are among the features that distinguish var. crus-galli from the obligate rice weeds E.crus-gallivar.oryzoides and *E.oryzicola* which are characterised by a more erect and stiffer habit. (Photograph: Luc Audenaerde).

#### ﻿ Echinochloa muricata

Taxon classificationPlantaePoalesPoaceae

(P. Beauv.) Fernald, Rhodora 17(198): 106. 1915.

876BE40E-FDA4-58A8-A345-5D8F06231D1E

##### Basionym.

*Setariamuricata* P. Beauv., Essai Agrostogr. 51, 170, 178. 1812.

##### Type.

Canada: Quebec Lac Champlain, s.d., *A. Michaux* s.n. (holotype: P-MICHX, isotype: US-80768).

###### *Echinochloamuricata* var. *muricata*

#### ﻿ Echinochloa muricata

Taxon classificationPlantaePoalesPoaceae

var. microstachya Wiegand, Rhodora 23(267): 58–60. 1921.

64E20BCF-6613-58EB-A372-DCF33EACAE02

##### Type (lecto-).

USA: New York, Tompkins Co., Ithaca, between Fall Creek, Inlet and city, waste soil, border of west marsh, open alluvial and marshy flats, 19 Jul 1913, *E.L. Palmer* 097 (GH). Image available at https://s3.amazonaws.com/huhwebimages/755E8AFFFFF6435/type/full/303931.jpg.

#### ﻿ Echinochloa muricata

Taxon classificationPlantaePoalesPoaceae

var. wiegandii (Fassett) Mohlenbr., Ill. Fl. Illinois (ed. 2) 396. 2001.

A05B47D0-5D88-59C1-976F-26981D785BBA

##### Basionym.

Echinochloapungens(Poir.)Rydb.var.wiegandii Fassett, Rhodora 51(601): 2. 1949.

##### Type.

USA: Oregon, Hayden Island, sandy roadside, *J.C. Nelson* 1974, 8 Sep 1915 (holotype GH). Image available at https://kiki.huh.harvard.edu/databases/specimen_search.php?mode=details&id=126740.

##### Remarks on *E.muricata*.

*Echinochloamuricata* is native to North America. Its status as separate from *E.crus-galli*, which was inadvertently introduced there long ago from Europe, was contested by Hitchcock (1920, 1935, 1950). Hitchcock (1920) rejected the separate status stating that he was unable to distinguish the two species based on the distinguishing features given by Fernald (1915). However, further studies by Wiegand (1921) and especially by Fassett (1949) confirmed the separate status of *E.muricata* (Gould et al. 1972). Probably, largely due to Hitchcock’s influential publications, a significant share of American authors have for decades combined native and introduced taxa under *E.crus-galli* in floras and weed-control publications (Maun and Barrett 1986). In the 21^st^ century, some researchers still refer to New World *E.crus-galli* – not to be confused with *E.crus-galli* introduced in North America from Europe – rather than using the name *E.muricata* (Aoki and Yamaguchi 2008). By now, however, molecular research has confirmed *E.muricata* as a separate species, clearly distinct from *E.crus-galli* (Claerhout et al. 2016; Mascanzoni 2018). This should put an end to the confusion that goes back to the days of Linnaeus, as it has been demonstrated that the type specimen of *E.crus-galli* in fact belongs to *E.muricata* (Crespo et al. 2020a).

*Echinochloamuricata* is a highly variable species, though less so than *E.crus-galli*. This, combined with its resemblance to *E.crus-galli*, has added to the difficulty for agronomists and botanists on both sides of the Atlantic to detect and correctly name its introduced populations. Early records of introduced *E.muricata* from France revealed morphologically very uniform populations (as *E.pungens* [Poir.] Rydb. var. microstachya [Wiegand] Fernald & Griscom; Deschatres et al. 1974). This resulted in identification keys that made it harder to correctly identify clearly deviating forms of *E.muricata* that had established in maize fields in Belgium (Hoste 2004).

The European populations of *E.muricata* exhibit only part of the variation found in the natural range of the species. So far, three morphologically distinct varieties have been recorded from Belgium and France. Echinochloamuricatavar.muricata, with larger spikelets, seems to occur only as an ephemeral alien (Hoste 2004). References to this variety in France require confirmation as they are probably based on misidentifications (Jauzein 1995; Tison and de Foucault 2014). The specimens with smaller spikelets recorded from Belgium are of two clearly different types, apparently with very few intermediates. The characteristics given in the key are mainly based on observations on European-origin plants. Specimens with unawned spikelets with strongly spreading bristles are assigned to var. microstachya Wiegand, and those with shortly awned spikelets with more appressed bristles to var. wiegandii (Fassett) Mohlenbr.; see Hoste (2004) and Bomble (2016) for illustrations of the inflorescences and spikelets. Genetic research on specimens collected from maize fields in Belgium has resulted in two clusters of *E.muricata* collections (Claerhout et al. 2016). From the study of the morphological features of three of these collections, we tentatively conclude that the two clusters C and D identified by Claerhout et al. (2016) correspond to var. wiegandii and var. microstachya, respectively (IH, unpublished data). Nonetheless, more genetic studies are needed to confirm whether the three morphologically distinct varieties are indeed genetically well-defined taxa. Both within and outside North America, forms of *E.muricata* with smaller spikelets have shown a stronger tendency to spread as weeds outside their natural range (Dore and McNeill 1980; Michael 2001).

*Echinochloamuricata* is a species of moist, disturbed sites. It is not an important weed of rice fields (Michael 2001, 2003) and in Europe it mainly occurs as a weed in maize fields (Hoste 2004; Bomble 2016).

#### ﻿ Echinochloa oryzicola

Taxon classificationPlantaePoalesPoaceae

(Vasinger) Vasinger, Fl. SSSR 2: 33. 1934.

DA457D89-987A-5DE4-8308-C22AA0DAD22D

= Echinochloa phyllopogon auct., non (Stapf) Stapf ex Kossenko in Botanicheskie Materialy Gerbariia Botanicheskogo Instituta imeni V. L. Komarova Akademii Nauk SSSR 8(12): 208. 1940.
= E. hostii auct. ital., non (M. Bieb.) Link, Hort. Berol. 2: 209. 1833.

##### Basionym.

*Panicumoryzicola* Vasinger, Trudy Prikl. Bot. 25(4): 125. 1931.

##### Type.

Vladivostok region, left bank of Santakheza, 4 km east of Lake Hanka, 23 Aug 1928, *A. Venzinger-Alexandrova* (lectotype, designated by Tzvelev 1976: 664, LE01010882). Image available at http://herbariumle.ru/?t=occ&id=15824&rid=image_0036250.

##### Remarks.

Although sometimes included in *E.crus-galli*, several features justify accepting *E.oryzicola* as a separate species. *Echinochloaoryzicola* is tetraploid (2n = 36), whereas *E.crus-galli* is hexaploid (2n = 54) (Yabuno 1966, 1981). The length of the embryo is a reliable feature to distinguish *E.oryzicola* from *E.crus-galli* var. oryzoides (which also has large spikelets) and from specimens of the very poorly defined E.crus-gallivar.hispidula. If carefully applied, the shape of the mature inflorescence and the length of the lower glume may help separate it from E.crus-gallivar.oryzoides. It is rather surprising that the seemingly distinctive feature of the length of the lower glume is not mentioned in Vasinger’s original description (Vasinger in Komarov 1934).

Yabuno (1966) distinguished two morphological forms of *E.oryzicola*: the F-form, in which the lemma of the sterile flower is flat and has a coarse surface texture, and the C-form, in which the lemma is convex, coriaceous and shiny. The latter form has only rarely been recorded from Southwestern Europe. Specimens with spikelets much too small for *E.oryzicola* but with a lemma that morphologically closely resembles Yabuno’s C-form have been recorded from Germany (as E.crus-gallisubsp.spiralis; Scholz 2002) and Belgium (IH, unpublished records).

The treatment of the rice mimics *E.oryzicola* and E.crus-gallivar.oryzoides in taxonomic and agronomic publications has been extremely confusing. In the past, the name *E.phyllopogon*, often without author citation and thereby adding to confusion, was used separately for each of the two taxa as well as for both of them together; see, e.g., the shifting interpretation in successive publications by Michael (1983, 1994, 2001) and Yabuno’s (1981) discussion of European *E.phyllopogon* as a synonym for *E.oryzicola*. *Echinochloaphyllopogon* is a very confusing name, whose identity has been recently summarised and discussed by Crespo et al. (2020a). Its basionym, *Panicumphyllopogon*, was described by Stapf (1901). The accompanying plate shows a specimen that seems to combine features of at least two species. It was said to have been collected by Arcangeli in rice fields near Pisa (Italy). Arcangeli’s herbarium is located in PI and FI, at least for the most part. A targeted search in the Arcangeli Herbarium (PI-ARC) did not yield any *Echinochloa* specimen collected in the rice fields near Pisa (comm. F. Roma-Marzio, 09.2018). In the Herbarium Generale of PI, there is a specimen labelled as *P.phyllopogon*, which was part of Flora Italica Exsiccata. The herbarium label states that this species was collected in Italy for the first time in Novara and that Stapf erroneously indicated it to be from Pisa. In fact, the species was collected by Jacometti near Novara but was originally, erroneously so, attributed to a collection of Arcangeli from near Pisa (comm. N. Ardenghi 10.2018). A lectotype for this name was designated by Kossenko (1940) based on one of Jacometti’s collections (K000958854; image available at http://www.kew.org/herbcatimg/638594.jpg). This collection includes both vegetative and flowering material that, according to P.W. Michael, refers to two different species. The non-flowering part, with very characteristic hair tufts at the junction of leaf blade and leaf sheath, was said to represent *P.phyllopogon* and was recommended to serve as (second step) lectotypification for that name (Michael 1983). However, the presence or absence of such hair tufts is a non-diagnostic feature that can be observed (although not so frequently) in various species of *Echinochloa*, including *E.oryzicola* and E.crus-gallivar.oryzoides. Since both these taxa occur in the Novara area in Italy, it is impossible to assign Stapf’s *P.phyllopogon* to one of these taxa. Therefore, it is a confusing name that should be abandoned. However, lectotypification of *P.phyllopogon* was effected later by Kossenko (1940) himself, though under the combination “E.phyllopogon subsp. stapfiana Kossenko”, a superfluous, illegitimate name that explicitly included the type of the species ( subsp. phyllopogon). Crespo et al. (2020a) argued this lectotype is to be followed; this made the later lectotype proposal by Michael (1983) ineffective. Consequently, *E.phyllopogon* should be included as synonymy of *E.oryzoides*, as suggested by Crespo et al. (2020a) and Martínez-Azorín and Crespo (2021).

**Figure 13.**
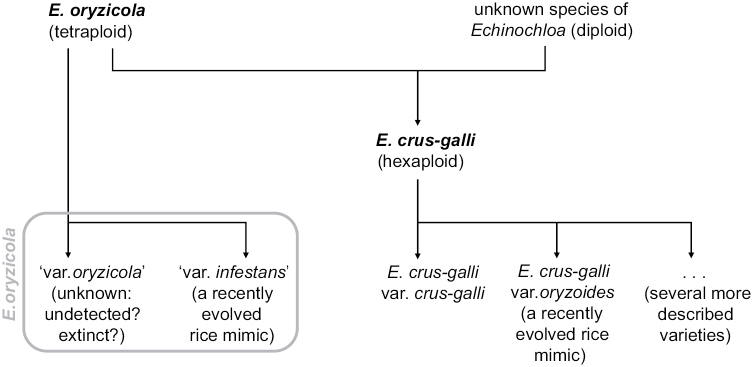
Schematic reconstruction of the evolutionary history of *Echinochloaoryzicola*, *E.crus-galli* and two rice mimics derived thereof. It is hypothesised that the taxon that today is called *E.oryzicola* has only recently evolved from a wild taxon that seems no longer to exist or has not yet been identified. In the absence of information on this original species, it is impossible to distinguish between a long-existing taxon (‘var. oryzicola’) and a recently evolved rice mimic (‘var. infestans’).

The separate status of *E.oryzicola* has been corroborated by molecular studies (e.g., Yamaguchi et al. 2005; Ye et al. 2014), although Yasuda and Nakayama (2019) have shown that relying solely on cpDNA may result in misidentification of E.crus-gallivar.formosensis as *E.oryzicola*.

Unfortunately, the structure of the tip of the fertile lemma, which clearly distinguishes *E.crus-galli* from *E.muricata* (Hoste 2004), has received little attention in studies on the weed flora of rice fields in Europe and Asia. In *E.oryzicola*, the tip more closely resembles *E.crus-galli*, although the line of tiny hairs is usually more difficult to see than in *E.crus-galli* (based on specimens from Italian rice fields seen by us; Fig. 4A).

### ﻿Taxonomy of *Echinochloa*: morphology, genetics and evolutionary history

Defined as “an ubiquitous plant, with variation you can’t get your teeth into, which clutters up herbaria” (Anderson 1952), the complex of *Echinochloacrus-galli* and a few closely related taxa fits the definition of a weed perfectly. In the decades after the publication of tentative keys for the annual and perennial species of the genus worldwide (Michael 1983), numerous studies have tried to solve the taxonomic problems relating to this genus. In general, these studies were mostly intended to give an overview of the species that occur in a restricted geographical area (e.g., in country floras) or to help find remedies to lower the impact of *Echinochloa* as noxious weeds in crops (such as rice and maize), which presupposes a correct identification of the taxa involved. Molecular studies covering a wider range of species are available without, however, linking genetics with morphology; see, e.g., Aoki and Yamaguchi (2008).

So far, *Echinochloa* has not benefitted from the recent revival of interest in botanical monographs, which has primarily been kindled by biodiversity and conservation concerns, especially in the species-rich tropics, rather than by hopes of improving the means to control economically damaging weeds (Grace et al. 2021). However, we believe that a worldwide monograph based on the integration of different scientific expertise including specimen-based taxonomy, genomics and phylogenetics (Muñoz-Rodríguez et al. 2019) is a prerequisite if we are ever to understand the complex taxonomy and evolutionary history and taxonomy of this genus. Once the evolutionary history is better grasped, it will become easier for weed scientists as well as the authors of regional floras to tackle the topics of interest.

The expression ‘evolutionary history’ here refers to more than ‘ancestry of a species’ as routinely used by biologists when describing ‘natural’ events. It also involves human history and the role humans have played, consciously or not, in the origin and evolution of plant species (Russell 2003, 2011). As for *Echinochloa*, a good understanding of what took place in Southeast Asia is essential in order to properly grasp the nature and significance of the diversity of forms displayed by the genus’ representatives in Southwestern Europe. In the latter geographical area, the species under consideration include a single introduced American species (*E.muricata*), the pantropical weed *E.colona* (native to the Old World, possibly including parts of Mediterranean Europe) and the complex of *E.crus-galli* and *E.oryzicola*, originally from Eurasia. Two additional cultivated taxa with non-shattering spikelets (*E.esculenta* and *E.frumentacea*) have both originated in Asia.

*Echinochloamuricata* exhibits a high degree of variation. Although within North America the distribution of the different forms has been altered as the result of human activities, such as land reclamation (Dore and McNeill 1980), the morphologic and genetic make-up of the species has most likely not strongly been affected by anthropogenic factors. The same can probably be said of polymorphic *E.crus-galli* in Europe. In Southeast Asia, the story is different and more complex. The result of a hybridization event between tetraploid *E.oryzicola* and an unknown diploid species, hexaploid *E.crus-galli* arose around 3.3 million years ago (Ye et al. 2014). *Echinochloaoryzicola* and *E.crus-galli* thus share a number of features, but the latter shows a wider range of morphological variation and ecological tolerances, which may be attributed to the added set of chromosomes (Yabuno 1966). Over time, the natural range of *E.crus-galli* has extended from East Asia to Western Europe, while the natural range of *E.oryzicola* apparently remained restricted to Southeast Asia.

Circa 10 millennia ago, *Echinochloa* spp., along with other wetland grasses such as rice (*Oryza* spp.), was gathered and processed for human consumption in China (Yang et al. 2015). *Echinochloa* fell out of favour when rice gradually evolved into a better-yielding crop (Chang 2000). It persisted, however, as a noxious weed and adapted in response to human activities such as the creation of rice paddies, hand-weeding and the timing of rice harvesting. Over time, *E.crus-galli* developed into a multitude of physiologically, morphologically and genetically different forms, displaying varied life histories in a range of habitat types (Yabuno 1966; Barrett and Wilson 1981; Yamasue et al. 1989; Fukao et al. 2003). As a result of unintentional selection, *Echinochloa* in rice paddies developed similarities with rice. Along with other features such as a more erect habit, rice mimics are characterised by green seedlings and a green culm base, having lost the anthocyanin pigmentation that is typical for E.crus-gallivar.crus-galli. Judging from the end result, it seems logical to conclude that *E.oryzicola* followed a parallel line of evolution. However, this raises a question. Regarding *E.crus-galli*, both the ancestor (var. crus-galli) and the evolved rice mimic (var. oryzoides) have been identified, yet in the case of *E.oryzicola* we apparently only know the mimic. Barrett (1983) distinguished between the strategies of the general purpose genotypes (such as var. crus-galli) and those of specialised biotic ecotypes (such as the rice mimic var. oryzoides). As for the specialised biotic ecotype *E.oryzicola*, no morphologically distinct generalist ancestor has been described. Barrett’s nomenclature reflects the shared morphological features of the rice mimics that occur in wetland rice paddies. His E.crus-gallivar.oryzicola included both an early-flowering hexaploid (*E.oryzoides* (Ard.) Fritsch = E.crus-gallivar.oryzoides) and a later-flowering tetraploid (*E.phyllopogon* (Stapf) Koss = *E.oryzicola*). Moreover, Yabuno (1966) described a rice mimic of *E.crus-galli* in upland rice fields; it shares the stiffer plant habit with the mimics from wetland rice paddies.

*Echinochloacrus-galli* is usually autogamous. When unconsciously transported around the world with rice seed, the introduction of morphologically different forms may, therefore, result in the establishment of seemingly quite distinct taxa (Barrett and Seaman 1980). This, in turn, may lure botanists into describing new species based on material that only poorly represents the range of variation in the area of origin, as illustrated by *Panicumoryzoides* Ard. (syn. E.crus-gallivar.oryzoides; Crespo et al. 2020b) and *P.erectum* Pollacci (‘*E.hispidula*’; Ardenghi et al. 2015), both based on materials collected in Italy.

Yamaguchi et al. (2005) stressed the poverty of sequence variations within a complex species such as *E.crus-galli*, despite the fact that the species shows a high morphological diversity, including domesticated forms, non-shattering weedy forms and shattering forms that mimic rice plants. The features of rice mimics, such as green culm base and seedlings, may be of limited value to taxonomists. In rice paddies, plants with red- or purple-tinged seedlings might again re-emerge now that herbicides have replaced hand-weeding. In a genus in which over the last 10 millennia, significant pre-existing morphological variation has been greatly increased due to close association with agriculture, accepting each seemingly well-defined form as a separate taxon may not lead to a satisfactory classification. For weed scientists, less-visible features related to ecological requirements, variation of the life cycle and development of resistance against herbicides may prove more relevant than morphological differences that once originated in a different co-evolutionary setting.

From this short detour into the evolutionary history of *Echinochloa* in Southeast Asia, one can conclude only that the study of the taxonomy of this genus in Europe requires a broader geographical scope. This should be coupled with the consideration of some questions that so far have been insufficiently addressed. The morphological and genetic variation of E.crus-gallivar.crus-galli within its extensive Old World native range is poorly documented, as are the interactions (occasional cross-pollination of usually autogamous plants) between populations of var. crus-galli and those of the derived rice mimics. As for *E.oryzicola*, in the absence of information about its non-mimic ancestor, its evolutionary history is quite obscure. Identifying the unknown diploid parent species that, together with tetraploid *E.oryzicola*, gave rise to *E.crus-galli* would help better understand the species complex of *E.crus-galli* and *E.oryzicola*, including ‘*E.glabrescens*’.

Embedding these questions in a larger project of a world monograph of *Echinochloa*, the outcome of the collaboration of experts in the fields of taxonomy, genomics and phylogenetics, would enhance our understanding of the affinities between weedy and non-weedy taxa, and between Old and New World species. Moreover, such a project could generate a great deal of knowledge about the evolutionary history of a group of plants that has undergone profound changes resulting from its interactions with humans in the course of the past millennia.

## Supplementary Material

XML Treatment for Echinochloa colona

XML Treatment for Echinochloa crus-galli

XML Treatment for Echinochloa crus-galli var. crus-galli

XML Treatment for Echinochloa crus-galli

XML Treatment for Echinochloa esculenta

XML Treatment for Echinochloa frumentacea

XML Treatment for Echinochloa muricata

XML Treatment for Echinochloa muricata

XML Treatment for Echinochloa muricata

XML Treatment for Echinochloa oryzicola
